# Developing Mental Toughness: Lessons from Paralympians

**DOI:** 10.3389/fpsyg.2017.01270

**Published:** 2017-08-03

**Authors:** Alexander J. Powell, Tony D. Myers

**Affiliations:** Sport, Physical Activity and Health, Newman University Birmingham, United Kingdom

**Keywords:** Paralympians, psychological, cognitive strategies, characteristics, post-traumatic growth

## Abstract

Mental toughness (MT) is a key psychological variable related to perseverance and success in performance domains. MT and its development has been explored across a range of contexts and across different sports, but no research to date has examined MT in relation to Paralympic athletes. We sought to understand the lived-experiences of mentally tough Paralympians, aiming to conceptualize MT in a Paralympic context and investigate its development. Ten Paralympic athletes were interviewed using in-depth, semi-structured interviews. The verbatim transcripts of the interviews served as the data for an interpretative phenomenological analysis. Three broad themes and several subthemes emerged in conceptualizing Paralympian MT: characteristics (determination, defiance, pragmatic, optimistic, resilient, self-belief and independence and autonomy), cognitions (normalization, sense of escape, non-acceptance of constraints, influence perception and connection) and cognitive strategies (rational thinking, goal setting, pain management and control). In understanding MT development, two broad themes and several subthemes emerged: formative experiences (challenge, classification, setbacks, critical incident, trauma and recovery, sustained commitment, development of mind-set and perspective during challenge, failure, and acceptance), and support and coping resources (social support and significant others, external shaping, social support, overcoming problems, social comparison and reflective practice). The findings suggest that Paralympians benefited from exposure to highly demanding situations in a supportive environment and this helped develop mentally tough characteristics and behaviors and individualized cognitive coping strategies. Our findings highlight the association between the adaptive development of personal characteristics by overcoming physical and mental setbacks over a sustained time period. Overall, the findings suggest that to develop mentally tough characteristics and behaviors, athletes in general could benefit from exposure to highly demanding situations in a supportive environment.

## Introduction

Mental Toughness (MT) is a quality that seems to be easily recognized by fans, coaches and players, yet remains far more elusive conceptually, being described differently in different contexts (e.g., see Crust, 2007; Gucciardi et al., 2015a) with no agreed operational definition. Perhaps this is because mentally tough behaviors are more directly observable than the cognitions, attitudes, and affect that accompany such behavior. For example, an individual who continues to persevere and achieve their goals in situations so adverse they would ordinarily be expected to falter and fail, would almost ubiquitously be described as demonstrating mentally tough behavior.

While MT appears unconvincing as a clearly defined psychological construct at present, there is some consensus regarding its nature. Most researchers consider MT to be a fairly stable disposition, albeit one that can change in the face of particular types of experience. The maxim ‘no pain no gain’ may be more than mere rhetoric when it comes to MT. Several researchers have identified the importance of hardship and adversity in the development of MT (Gucciardi, 2010). Current perspectives suggest that MT represents a collection of personal resources that are salient for goal-directed behavior despite varying degrees of situational demands (Gucciardi et al., 2015b). In terms of MT development, research also seems to support intuition and anecdotal evidence that athletes must be exposed to, rather than shielded from, demanding situations, challenges and adversity (Crust and Clough, 2011; Collins and MacNamara, 2012).

Recent research seems to support the importance of exposure to adversity. Sarkar et al. (2015) explored the beneficial effect of adversity with 10 Olympic gold medalists. The Olympic champions suggested that adverse experiences initially led to trauma, but rather than eliciting maladaptive behavioral responses, the intense negative emotions were used to fuel the athletes’ future effort and application. Another study exploring the adversity and growth-related experiences of Olympic swimmers found that by adopting transitional related strategies, the athletes ultimately thrived in the face of adversity, and flourished as performers and human beings (Howells and Fletcher, 2015). This type of transitional response to adversity is not unique to Olympic champions, and is considered to be an important formative experience for talent development (Savage et al., 2016). The primarily sports-based traumas described were viewed as negative experiences but were retrospectively seen by the athletes as having a positive impact. This study provides further evidence that traumas or memorable challenges are important to an athletes’ development. Research suggests that hardship may be important in developing performers’ potential as athletes, and it is important to determine why some athletes flourish and others fold under similar circumstances. Findings such as these have led to the idea that socially contextual factors that influence and impinge upon the lives of the athlete must be taken into account when exploring MT development (Crust and Clough, 2011; Weinberg et al., 2011).

Psychology has seen a shift from people viewing stress and trauma in a negative light and has also explored the positive effects that can be experienced following highly stressful or traumatic events. Post-traumatic growth (PTG) is one of the terms used to describe such experiences (Tedeschi and Calhoun, 1995). The research suggests it is not necessarily the trauma that encourages PTG, but the cognitive processing and affective engagement following an experience that leads to perceptions of positive change and/or learning (Tedeschi and Calhoun, 2004a). Given the relationship between overcoming traumatic events and the ability to cope successfully with future stress, it may be beneficial for structured development programs to include deliberate challenges that require athletes to face and overcome difficult situations. Not surprisingly, studies specifically investigating MT in sport have predominantly focused on sport related trauma and challenge (Howells and Fletcher, 2015; Sarkar et al., 2015; Collins et al., 2016). Nonetheless, researchers of non-sport related trauma have documented remarkable levels of resilience when people are exposed to bereavement or potentially traumatic events at some point in their lives, and yet they continue to have positive emotional experiences and show only minor and transient disruptions in their ability to function (Bonanno and Mancini, 2008). It has been suggested that traumatic events including serious illness, injury and bereavement almost always produce lasting emotional damage but not everyone confronted with such events reacts the same way with varying responses whereby some people are debilitated and others are only minimally affected and then gradually recover (Bonanno and Mancini, 2012). Furthermore, gaining a psychological benefit after experiencing a negative event is believed to be a common occurrence and will act as a form of ‘future proofing’ against subsequent events (Bonanno, 2008).

One group of athletes that exemplify PTG is Paralympic athletes. Para-sport allows people with disabilities to achieve extraordinary heights of functional capability and in many ways has become an elite sport, with increased training intensity, sports performance and improved training methods. Paralympic athletes constantly have to deal with: sport overuse, risk behavior, functional limitations, psychological stressors, normalized pain, health hazards, and unequal prerequisites (Fagher et al., 2016). Relative to able-bodied athletes, very little is known about the mental skill use of Paralympic athletes, as the majority of research has been conducted with non-elite disability athletes (Martin and Malone, 2013). However, it has been reported that athletes with disabilities demonstrate stronger resiliency and self-efficacy skills than able-bodied athletes (Martin et al., 2011). Perspectives taken from Paralympic athletes with varying injuries may provide an insight into the previously unidentified qualities and strategies.

It can be assumed that athletes with disabilities have had to overcome more ‘trauma’ than non-disabled counterparts, particularly in their non-sporting life. Typically, research has compared athletes with disabilities to athletes without disabilities, and athletes with acquired disabilities to athletes with congenital disabilities (Dieffenbach et al., 2009). One rationale for these comparisons is that because many athletes with disabilities have experienced a major life trauma, they may differ from athletes without disabilities on various coping skills, mood states, or other important self-perceptions (Martin et al., 2011). Dieffenbach et al. (2009) found that athletes with disabilities report spending less time engaging in mental training and have fewer opportunities to formally learn mental skills. It is conceivable that the potential lack of learned mental skills means that athletes with disabilities may rely on mental skills developed without formal input to facilitate training and performance (Martin et al., 2011).

There is currently a lack of knowledge of the psychological strengths and MT of Paralympic athletes. The lack of clarity and consistency regarding MT is due in part, to both a lack of conceptual maturity and literature on its conceptualization. This alludes to a gap in the MT literature and warrants further study. Such knowledge could confirm and prioritize the importance of factors already identified, as well as uncover additional factors necessary for the development of MT and exceptional performance in areas previously not investigated. We feel that research needs to be extended by taking the perspectives of these groups into account to establish a broader yet more nuanced conception of MT and it development. As such, the overall, the aim of this study was to investigate MT and its development in Paralympic sport by exploring the perceptions and experiences of Paralympic athletes.

## Materials and Methods

### Methodology

Given the aims of the study, a qualitative approach was considered the most appropriate. This is consistent with suggestions to employ in-depth qualitative approaches to study MT (Crust, 2007; Weinberg et al., 2011; Gucciardi et al., 2012), something that seems more pertinent given the novel participant group involved. However, given the aims went beyond just exploring the perceptions and experience of Paralympic athletes to also consider its development, it was important to take an ontological and epistemological stance consistent with these aims. Namely, a position where individuals are able to sensibly share their experiences in a meaningful way, and others are able to connect with those experiences and learn from them.

Critical realism satisfies this in taking a realist ontology, yet a constructivist epistemology (Bhaskar, 1978); that is, accepting stable and enduring features of reality exist independently of human conceptualization, while considering there is no unmediated access to this reality (Al-Amoudi and Willmott, 2011). A method consistent with this philosophical position is Interpretive Phenomenological Analysis (IPA; Smith et al., 2009). The interpretive approach used in IPA is theoretically rooted in critical realism and the social cognition paradigm. The social and cognitive connection in IPA provides a sufficient platform for the researcher to gain an understanding of mental processes (Seidman, 2006). IPA was therefore employed to explore Paralympians’ perceptions of MT and how they felt their own MT developed. Besides being consistent with a critical realist philosophy, IPA was considered appropriate as it is concerned with the importance of the ‘life world’ or lived experience of individuals with the researcher providing an interpretation of the participant’s interpretation (Smith et al., 2009).

### Participants

In accordance with IPA guidelines (Smith et al., 2009), a convenience sample of Paralympic athletes was initially used, from which a purposeful sample of athletes identified as demonstrating MT by other people in the sport, were recruited. The group included athletes from a range of sports with a range of experiences. The sample comprised of 10 Team Great Britain Paralympic athletes (nine male and one female) who have collectively won 6 gold and 5 bronze Paralympic medals. Participants met the inclusion criteria by competing at either one or more Paralympic games (i.e., Athens 2004, Beijing 2008, London 2012, and Rio 2016), and identified by other elite athletes in the sport as regularly demonstrating mentally tough behaviors. The age of the participants ranged from 19 to 44 years (*M* = 27.9, *SD* = 7.1) and a range of individual and team sports were represented: athletics, water sports, swimming, racket sports, field sports, and court sports.

The participants varied in the International Paralympic Committee (IPC) Athlete Classification Code (Tweedy et al., 2014). The ‘Classification’ is a defining feature of Para-sport; it is defined as grouping athletes into Sport Classes according to how much their impairment affects fundamental activities in each specific sport or discipline. The sample was made up of athletes that have acquired disabilities as a result of accident or disease after birth (*n* = 5) or congenital disability (*n* = 5). Given concerns about participant confidentiality, any specific details that might compromise their anonymity are excluded.

### Procedure

Participants meeting the inclusion criteria above were invited to participate through personal contact, either directly or through gatekeepers. The study received ethical approval from the University Ethics Committee. The participants were given an information sheet prior to the study to familiarize themselves with the aims, objectives, and procedures of the research. Once participants were satisfied with this information, signed informed consent was given. Pseudonyms have been used to preserve anonymity throughout the article.

### Data Collection

In accordance with the majority of IPA studies the data were collected via semi structured interviews (Smith et al., 2009). All participants were involved in a confidential interview conducted by the first author that lasted between 30 and 55 min, which is normal for IPA interviews (Smith et al., 2009).

To explore the perceptions and experiences of the Paralympic athletes’, a semi-structured interview guide was designed by the authors, taking previous research into consideration and being mindful of current gaps in the literature. Smith et al. (2009) stress the importance of formulating open questions, which do not lead participants toward particular responses, yet at the same time remain open to changes in the sequence and questions in order to follow up the answers given.

The first author conducted all interviews with an effort made to develop rapport with interviewees via appreciation of their history, achievements and development experiences. The interview guide was split into three broad categories: introductory questions and perceptions of how MT is conceptualized in Paralympic sport; experiences of MT and situations requiring MT (e.g., “What makes you mentally tough and in what situations would you say you are most mentally tough?”); the perception of MT in other Paralympic athletes (e.g., “Can you tell me about a particular athlete that you have worked with that you feel consistently demonstrates MT?”); perceived development of MT (e.g., “Why do you think they are mentally tough, and how do you think they have developed MT?”).

Prior to the data collection phase, one pilot interview was conducted with a professional staff member working in Paralympic sport to assess the efficacy of the questions. As a result, some questions were modified or adapted. All the interviews were captured using a digital audio recorder and transcribed verbatim (Kvale, 2007).

### Data Analysis

Under IPA the objective is to interpret the meanings, rather than measure frequency of any given ‘type’ of response. The analysis took place in the four stages. Following verbatim transcription, the researchers read and reread the data. This involved the researchers immersing themselves in the original data. This process was repeated several times enabling immersion into the participant’s world. This approach is consistent with the chosen methodology, because in phenomenology the participant is considered the “expert” and it is the meanings he or she associates with his or her experiences that are of interest (Seidman, 2006; Smith et al., 2009). The second stage involved the first author looking for themes in the first set of participant data. The initial step involved the interview transcript being split into three columns. The original interview text was placed in the middle column, exploratory comments in the right hand column and emergent themes in the left hand column. The exploratory comments included descriptive, linguistic and conceptual areas. The descriptive comments focused on describing the content of what the participant has said. The linguistic comments focused upon exploring the specific use of language by the participant. The conceptual comments focused on engaging with the transcript at a more interrogative and conceptual level, connections were made between participants’ actual statements and the first author’s interpretations (Smith et al., 2009).

Combining the original transcript with the exploratory comments enabled the researchers to be further immersed in the participant’s life world and engage fully in deep data analysis. The third stage aimed to connect the themes (Smith et al., 2009). Once all 10 transcripts had been subjected to IPA in their entirety, a list of emergent themes reflecting the participants’ perceptions of MT attributes and MT development was created. The fourth stage involved these themes being connected to each other based on similarities and apparent interrelationships. Once a set of themes had been established, the next step involved clustering themes together. The themes were checked with the transcripts to ensure that the connections included the primary source material (i.e., the actual words of the participant) (summaries of which are presented in the results section in **Tables 1, 2**).

**Table 1:** Summary of themes and examples of raw data extracts for MT conceptualization in Paralympians.

Mental Toughness	Number of participants	Selected quotes from participants
**Characteristics**		
Determination	10	“… it’s just like, what do you mean limited, were doing this OK… They are almost offended to the point where they are like; of course I can do it.”
Defiance	9	“I just phone the consultant and say this is broken… in and out…if they can’t, then I will not have the surgery, I’ll just continue… it doesn’t really bother me.”
Pragmatic	10	“As disabled athletes we do not see it as being harder…if you are in a chair or a limb missing then obviously… there are more obstacles.”
Optimistic	9	“I mean that willingness to overcome that mass of adversity that they have suffered and just take the opportunities that have come”
Resilient	9	“…I can think of three or four guys that have been broken by the classification system.”
Self-belief	10	“…we would not say that we are superhuman, we do not want to be symbolized as being special, the whole idea of doing sport as Para-athletes is that you want to be the same level as Olympic athletes”
Independence and autonomy	10	“…when I am in the chair, I am the best in the world… so for me it is freedom and independence and it is something I do not need help doing.”
**Cognitions**		
Normalization	10	“…because they do not want to be defined by what happened … they just want to be like everybody else and do not want to be that horrible story.”
Sense of escape	9	“…so it is probably one of the only times where I don’t feel disabled because I feel like I am just an athlete playing a sport that I love”
Non-acceptance of constraints	8	“…I will find a way around it, Paralympic sport is rife for it, you can’t be lifting that leg…you can’t be doing this because of your classification…”
Influence perception	9	“…people still think ‘Arrrr, they are having a go’… I’m thinking, ’no’, Paralympics’ isn’t inspiration, it’s trying to prove disability isn’t a bad thing’
Connection	10	“…I wanted to represent GB. I knew that from the very early stage and that helped me with my injuries my focus and my rehabilitation.”
**Cognitive strategies**		
Rational thinking	10	“…sport is a fairly simple thing, sport is not life or death… so breaking your back or losing a leg, sport will never be difficult in that context.”
Goal setting	10	“I think it is about dealing with failure, dealing with setbacks, setting goals and not being happy…and thinking they’ve made it”
Pain management	10	“…have the ability to overcome pain and you can remove those thoughts of pain at the right point when the pressure is all on you.”
Control	10	“…so when you get that higher level of anxiety… being pushed by adversity over and over again, it’s a way of bringing it back to real life”

**Table 2:** Summary of themes and examples of raw data extracts for MT development in Paralympians.

Mental toughness	Number of participants	Selected quotes from participants
**Formative experiences**		
Challenge	10	“…I was chasing the guys in my classification that were 20 years older and were twice my size… I loved that challenge, I was almost enjoying failing.”
Classification	9	“…those pressures…anxieties around classification, physical and mental screening then to be poked and prodded…medical records scrutinized”
Setbacks	10	“…we were battling classification and when they changed his classification, the parts that he could do well, he could no longer use”
Critical incident	10	“…and I have heard athletes say it themselves because that event redefined who they were and it gave them purpose…”
Trauma and recovery	10	“…the whole realization started to build on my shoulders and I did go into an almost state of depression because I didn’t know what to do.”
Sustained commitment	9	“…just learning to roll from your back to your side took 6 weeks Errmm… So it would be quite easy to say, I can’t be bothered with this, I’ll just give up“
Development of mind-set and perspective during challenge	9	“…it’s never easy but you have a perspective and think that this is not that bad, I know how bad feels and looks like and this is nowhere near it.”
Failure	10	“Losing helps you develop mental toughness… they lost one race once by a classification and that athlete has broken down in tears.”
Acceptance	9	“I have just accepted that I am forever with pain…. I know that I can deal with pain and I can find ways to shut off the pain and keep going.”
**Support and coping resources**		
Social support and significant others	10	“…you need to try and be strong for other people, my family has suffered a lot with my injury, so I cannot be getting upset about stupid little things.”
External shaping	9	“…they didn’t want to accept that I was in a wheelchair, they didn’t want to accept I was different, that wheelchair made me feel like I was different…”
Overcoming problems	10	“One thing that I have found with Paralympic athletes, they all have different challenges set by doctors, nurses and physios.”
Social comparison	9	“…being surrounded by varying degrees of disability athletes all striving to be the best in the world, I’d say that it’s easier to understand where you’re at”
Reflective practice	10	“…OK, it’s an inconvenience, I think that a lot of people would prefer not to have it but I would not be in this position… if I didn’t have my disability”

### Addressing Trustworthiness

Several approaches were employed to optimize data trustworthiness (Smith et al., 2009). The criteria for enhancing the quality of work included the following: the worthiness of the topic, the significant contribution of the work, and rich rigor (e.g., developing a sample appropriate for the purpose of the study and generating data that could provide for meaningful and significant claims) (Smith et al., 2009). Bracketing, investigator triangulation and member checking were used to check the trustworthiness or goodness of the data.

A reflexive diary was kept in order to reduce the influence of possible bias created by previous interactions and personal experiences, this bracketing was implemented during all stages of the research. Firstly, the researchers identified their own assumptions held about MT and Paralympic athletes and ongoing judgments about the data and interpretations of these. The bracketed assumptions allowed the researcher to consciously and regularly check if they were imposing any meanings on the data and to observe if any other meanings appeared. The second engagement was the hermeneutic revisiting of data and the evolving comprehension of it in light of revised understanding of any aspect of MT (Fischer, 2009).

In addition to highlighting the text, researchers discussed their assumptions, explored alternative explanations and interpretations as these emerged in relation to the data. Seidman (2006) suggests this cyclical process allows the researchers to become aware of their own preconceptions, question analytical decisions and bracket them to engage with the participants’ meanings, and then return to an altered set of conceptions. These techniques raised the researchers’ self-awareness and ultimately added to the quality and validity of the data analysis.

Member checking was conducted by offering the participants the opportunity to review and discuss the researchers’ interpretations of their statements (Kvale, 2007). The first author contacted the interview participants via email and requested feedback on what the researchers had considered the quotes to signify and the context of the results section in which they would appear.

Finally, quotations have been included in the results section to illustrate themes and to allow readers to form their own interpretations. Pseudonyms were used to protect participants’ identities and also to identify whether the participant has an acquired disability (Acq) or congenital (Con).

## Results

**FIGURE 1:**
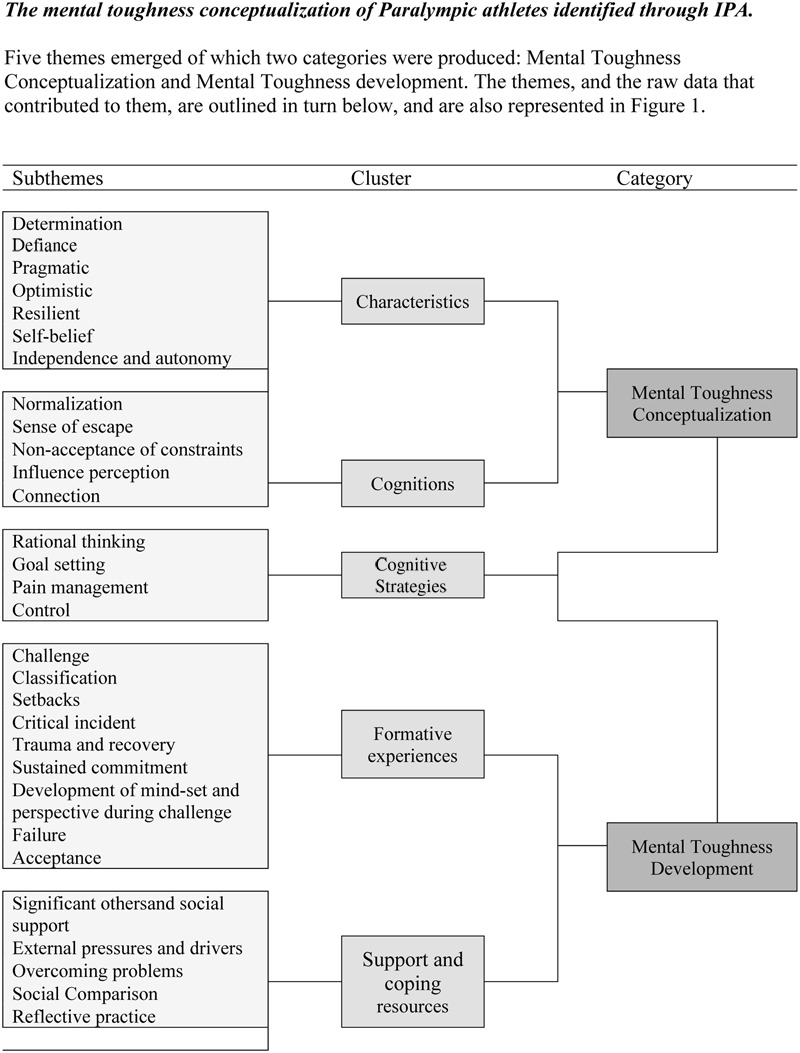
Perceptions of mental toughness conceptualization and development reported by Paralympic athletes.

Themes were interpreted from the data and formed natural clusters, which represent the grouping at a more theoretical level (see **Figure 1**). Summaries of these clusters and themes are presented in order to provide the reader with more transparency by which to judge our analysis (see **Tables 1, 2**).

### Conceptualization of MT in Paralympians

This category was created from data regarding the overall conceptualization of MT. These fell into three key themes: characteristics, cognitions and cognitive strategies.

The overall perception of MT across participants is highlighted in the following examples:

“…because on the whole Paralympic athletes have suffered tremendous adversity in some way or another, through an injury or born with a disability that has really affected them. So they have already had an uphill battle at some point in their life and they have overcome that by taking an opportunity to play sport… it is very inspirational how people get over those traumatic experiences” Rich (Acq).

“…it is having to overcome lots of different barriers throughout their lives… So they do not just have to deal with the actual sporting element of it, but it’s the preparation, the training and lifestyle choices, the perception of disability when it comes to society. So they have got to deal with that a lot more barriers before they even take up sport and then I think when they get into sport, being a Paralympian there are even more barriers put in place… different classifications, different rule changes” Eddie (Acq).

### Characteristics

To inform MT conceptualization, it is important to elicit the distinguishing behavioral characteristics and qualities that define a mentally tough individual. It is also important to recognize the psychological characteristics and also to understand what they allow the athletes to do *in situ*.

#### Determination

A key characteristic suggested by the Paralympians, is the requirement and demonstration of high levels of determination. The accounts demonstrate a tenacious approach to completing tasks. It is noticeable that Dan associates his determination levels and how he perceives that he will be viewed by others.

“…they almost appear indestructible no matter what is put in their way, they will just find a way to deal with it, and often they would just do it off their own back, it is their own energy that they are putting in” Rich (Acq).

“…they wouldn’t ever mention the fact that they have got limitations in their disability and they would never mention… it’s just like, what do you mean limited? The athletes were like, limited? We’re doing this OK… they are almost offended to the point where they are like of course I can do it” Dan (Acq).

#### Defiance

Furthermore, the Paralympians described the importance of being able to sustain effort and continue to perform whilst experiencing difficulty. Both accounts below refer to experiences of pain management with Jamie demonstrating an appraisal of pain that could be perceived as ‘unhealthy’ by disregarding medical processes in the pursuit of personal goals.

“…it is understandable that they have had to go through a lot more pain in some ways naturally more than others and it brings on a sense of pain threshold and you’ve got to push through it…that is the MT to be able to be screaming inside and quiet on the outside” Pete (Con).

“…I have had five lots of surgery in 2 years….Up to Rio…I just phone the consultant and say I need this surgery, this is broken, they book me in and I give them 24 h to get it done, in and out to get straight back into training… and if they can’t do that I’ll not have it done, I’ll not have the surgery, I will just continue and then it breaks, it does not really bother me” Jamie (Acq).

#### Pragmatic

This characteristic related to a pragmatic approach to solving problems rather than being constrained by fixed ideas or beliefs of others. Eddie views problems as personal challenges and ultimately attributes personal success to overcoming them, for this reason he applies extended effort to overcome challenging situations in any way possible.

“I hated being told no, no, you cannot do this, I needed to prove people wrong, and I am going to do everything I possibly can to do it, if you tell me I cannot do something, I will find a way around the system to do it… Paralympic sport is rife for it, you cannot be lifting that leg, you can’t be doing this because you are in this classification, and you are not allowed to do this” Eddie (Acq).

“…in the Paralympics when they’re trying to make it competitive and minimize classifications, but with multiple disabilities the spectrum just grows between those in the class…. You have just got to be in that mind-set to continually go out and look for solutions to problems and not just allow it to be in front of you, I will find a solution and nothing is going to stop me” Eddie (Acq).

#### Optimistic

The perception that participants had of a mentally tough Paralympian was one that interprets situations and events optimistically, so that in some way negative outcomes did not appear to be fully comprehended. This characteristic was associated with overcoming adversity and belief that future situations will unfold optimally.

“…that willingness to overcome that mass of adversity that they have suffered and just take the opportunities… and it is just being able to separate things up in their life, so they can separate things in their mind… they can take that away from that moment and then embrace the moment that they are in now… looking at them you would never think that they have suffered from the things that they may have done” Rich (Acq).

“…they seize those opportunities, they do not just stay in their comfort zone…wallow in self-pity and think that the world is against me, they take a step out of their comfort zone and take up an opportunity… that is probably what people see on TV when they see somebody who is missing a leg or an arm or something…and all of the stuff that they get through and they still managed to go to Paralympic games” Rich (Acq).

#### Resilient

Resilience was a reoccurring term used in both the personal accounts and external interpretations of others. These perceptions related to experiences of sport and non-sport adversity (competition/training or major life events) and the potential positive adaptations.

“…someone like that, it amazes me and I think that if my boyfriend was killed, I would probably just sit down and say right, life is over… I think I would lose my confidence and then just wouldn’t know how to get on with life, never mind boyfriend killed, my life ruined and I cannot do anything for myself any more. It is amazing how she has picked herself back up, and got her confidence back and is just so independent and at the same time does not have a chip on her shoulder” Emma (Con).

“…I can just carry on…it’s just like if there is a really negative environment around me, it just doesn’t influence me, where as some people really get drawn into it. I don’t know what it is that is different about my brain and why it perceives things and can just switch off…. I don’t know why that is because I have never had any sort of like therapy or any of these sort of things, I have never, I have just got on with it (laughing), and that has been my way of dealing with it, I don’t even notice now” Dave (Acq).

#### Self-belief

Self-belief and high levels of self-confidence emerged as important when dealing with challenging moments and developing skills, abilities and behaviors associated with MT.

“Redefining my level after my injury and say right, I can’t do those things any more but I can do these things, being confident with that and making the most of it… I do think that I am stronger now the since my injury. I think I was quite strong willed before but now I think that I am a lot more stronger…I won’t get as annoyed about it or as upset about it and I will just take it in my stride and realize that’s just, that’s just life and you have got to try and stay positive and get on with it. Rich (Acq).

#### Independence/Autonomy

Independence and autonomy were considered to be associated with improved psychological functioning and motivation. Similarly, participants suggested athletes who demonstrated lower levels of MT had expectations of entitlement and were more reliant on external resources.

“…in the Paralympic world you meet a lot of athletes who feel like the world owes them something because of their accident, they feel like this has been taken away so now the world has to give me this gold medal, they have to give me this achievement and unfortunately the world does not grant wishes and that is not what it is therefore, it is their fate to work hard and make your own way in life” Emma (Con).

“A lot of athletes that were maybe born disabled and have been wrapped up in cotton wool, they just expect everything to be done, and you’re thinking do you not want independence to be able to get in your own chair or put your own blade” Emma (Con).

### Cognitions

This theme emerged due to participants emphasizing the mental actions or processes of acquiring knowledge and also understanding it through thought, experience, and the senses. The following sections highlight the most salient cognitions associated with MT in Paralympians.

#### Normalization

Numerous cognitions, behaviors and attitudes seemed to be motivated by the desire to demonstrate a ‘normal’ state and existence. The process of normalization is embedded in both sporting and non-sporting environments.

“…they always seem to put it behind them and not make it the existence of their living… because they do not want to be defined by what happened, I have heard some absolutely horrible stories but you have to kind of coax it out of them…they just want to be like everybody else and do not want to be that horrible story” Emma (Con).

Moreover, Paralympic athletes demonstrate affinity with Olympic athletes because of the shared and comparable elite sport levelness, characteristics and achievements.

“…but we do not want to be seen as being different, because that might see our sport being perceived as a harder one or, not a level playing field, we do the same training the same racing as anybody in the Olympic program… the only thing is that we have to do it a different way… we do not want that divide, I think having names put on us, provides that divide and obviously Olympic athletes are not described as ‘Super Human’ because they are just athletes, and we are just athletes, we are no different to them” Jamie (Acq).

#### Sense of Escape

Being immersed in sport appears to provide a sense of escape from disability by engaging in a highly satisfying and valued activity.

“…it’s one of the times where you don’t feel disabled if that makes sense. It is quite funny because I know, obviously we are playing a Paralympic sport a disability sport, and obviously playing it in a wheelchair, but it is a different feeling because, I am playing proper sports, I am playing rugby, the same way that I would have played football… So it is probably one of the only times where I don’t feel disabled because I feel like I am just an athlete playing a sport that I love” Tom (Acq).

#### Non-acceptance of Constraints

A refusal to accept limitations or restrictions was considered vital by Paralympic athletes. Acknowledgment was made for the requirement of rules and regulations but a determined and pragmatic approach was deemed necessary to remove any limiting factors of personal classification.

“…if you tell me I cannot do something, I will find a way around the system… Paralympic sport is rife for it… you cannot be lifting that leg, you can’t do this because you are in this classification, you’re not allowed to do this…So me and my Dad came up with this stupid contraption to build a throwing leg that we just strapped to the side of the frame with a foot attached to the floor and I would put my leg inside this socket, I could never walk around on it but the rules stated that you have to have one foot in contact with the floor, it didn’t say that it had to be your foot, it didn’t say anything about how it was strapped to you” Eddie (Acq).

#### Influence Perception

A significant driver emerging from the data related to perception that enduring physical training and sustained effort is associated with changing perceptions. Paralympians suggested they are able to forge their own identity, raise awareness and influence the behaviors of others.

“…with the Paralympics, the word inspiration is used a lot and actually, OK, some of the stories you can draw some inspiration…but people still think ‘wow they are having a go, that’s really cute,’ and then I am thinking, ‘no’! So to Paralympic athletes, it is not inspiration it is trying to prove something, prove that disability is not a bad thing… I would not be in the position I am today if I didn’t have a disability” Emma (Con).

“…I think the public make out that it is special. I know there have been issues when they came up with the ‘Super Humans’ logo during the Paralympics period… but we would not say that we are superhuman, because we do not want to be symbolized as being special because the whole idea of doing sport as Para-athletes is that you want to be the same level as Olympic athletes. I think having names put on us, provides that divide and obviously Olympic athletes are not described as ‘Super Human’s’ because they are just athletes, and we are just athletes, we are no different to them” Jamie (Acq).

#### Connection

John explains that the connection he feels within his team environment produces mentally tough behaviors, expectations and standards.

“…it is all about being noticed and noted by other people, but it’s about what we want them to notice with regards to our behaviors and our effort. It is something that brings us together as a unit and it encourages you to live by those values” John (Con).

Below is Jamie’s reflection on an athlete with a congenital disability who had not previously been immersed in a ‘connected’ disability sport performance environment. The extract demonstrates both the closeness and acceptance inside the group.

“…he hasn’t hung around with any disabled people before and he was really uncomfortable in that situation, we were all talking and ‘Crips’ and all other sorts of words were banging around because that is what people say when they are taking the piss out of people’s disabilities… and he found that really hard to handle. But you know you would think that he would be okay with it because he was born that way, you would think that is normal for him, but he could not handle it in that environment with disabled people” Jamie (Acq).

### Cognitive Strategies

This theme emerged due to a number strategies being described and associated with mentally tough behaviors. Participants applied individual and personalized plans of action designed to achieve overall MT in demanding situations, the most salient strategies are discussed below.

#### Rational Thinking

The thinking patterns, reason and use of logic applied in demanding situations were recalled. The accounts below describe logical appraisals, comparisons and also the development of this strategy through exposure and experience.

“…I remember when I first got injured because there are two paths that you can take, one where you sit down and be miserable and there is a path where you excel and it is your choice, obviously it is something that sort of happens” Jamie (Acq).

“…I feel like I am massively confident and I am able to deal with most situations, but probably the first year that I competed on the world stage, I didn’t have these attributes per-se, I didn’t have what I have now, like the MT… I just couldn’t put up the walls like the rational thinking and everything else and being like this is what I have got to do and I just fell apart at the first world’s (competition). I just massively failed compared to what I should have done, and couldn’t cope with it at all” Dave (Acq).

#### Goal Setting

The process of goal setting was used in varying situations from overcoming adversity and injury to performance related targets.

“…so I think it’s about dealing with failure, dealing with setbacks, setting goals and not being happy, not being too happy and thinking that they have made it, it’s thinking OK, I have achieved that, what is the next thing that I can achieve and setting goals” Rich (Acq).

“…so I thought where is my life going and then obviously I was introduced to wheelchair Rugby…I knew from then that that is what my main focus in life would be, and I wanted to be the best. I wanted to be the best in my classification, I wanted to represent GB. So I knew that from the very early stage and I think that that helped me with my injuries my focus and my rehabilitation because I came to believe that this was what I wanted, I put all of my time, effort and focus into it” Tom (Acq).

#### Pain Management

All athletes reported using personal strategies to alleviate the effect of pain during training and performance. Commitment to the sport is reported as the motivation for enduring the discomfort.

“I guess every Paralympian feels pain somewhere, so for me sat in the chair, I sit on my legs and I have full feeling in my legs so my legs go numb really quick and that gives me loads of problems with trapped nerves...but to me it hurts just walk anyway so what is the difference? It doesn’t really matter because I’m doing something that I enjoy, yes it hurts a little bit but it will recover and it will be fine” Emma (Con).

“…I have had an injury and I have had to be in the gym every day for 3 weeks doing something called occlusion training, which was very mentally tough, it is occlusion training where you cut off the blood to one of your limbs and then you just go until you fail and you have just got to keep telling yourself, just keep going, just keep going and 3 weeks of that, without doing normal training or anything, that was really quite mentally challenging” Phil (Con).

#### Control

When exposed to demanding situations, the Paralympians described various personal strategies to control their responses.

“…So when you get to that higher level of anxiety, you know, being pushed by adversity over and over again, it is a way of bringing it back to real life, such as breathing techniques, imagery techniques, reflection techniques just so the athlete starts to learn how to manage all of those different thoughts and feelings, so they can almost be, mentally tough, just by taking themselves out of the situation and concentrating on breathing and centring” Eddie (Acq).

### Development of MT in Paralympians

#### Formative Experiences

To inform MT development, it is important to understand the necessary experiences that athletes require. The most salient experiences and examples are presented below.

#### Challenge

It was perceived that challenge is present everyday both in a sporting and non-sporting context. Challenge was readily accepted and was interpreted as a positive opportunity rather than a threat.

“… mental toughness is the challenge of everyday life, so for me it is freedom and independence and it is something I do not need help doing… MT is in everyday life, if you compare me pushing my chair, if I stood up and walked through that door it would take 300 times more energy than an abled bodied person so actually for me it is trying to balance the fact that I am constantly tired from what I have to do” Emma (Con).

“… I was chasing all of the guys in my classification that were 20 years older than me and they were twice my size, and I loved that challenge, I was almost enjoying failing just to see how close I can get, creating those milestones like right, now I’m only 30 cm away, 20 cm away, 10 cm away and then when I actually did it, I kind of I realized it was all worth it” Eddie (Acq).

#### Classification

The fundamental and complex process of classification has been described as a source of anxiety and potential adversity. This is due to uncertainty and the impact the disability classifications can have on their ability to perform or even compete.

“…so just being able to deal with all of those pressures, all of those anxieties around classification… to be put through a rigorous, physical and mental screening and then to be poked and prodded by different physios and doctors, having all of your medical records scrutinized, just to put you in a classification is quite a daunting thing” Eddie (Acq).

“…changes to classification or whether or not an athlete is eligible to compete, their career could be should cut short by the ruling as to whether or not they are eligible for class, classes are removed from the program because there aren’t enough athletes…, so it could be entirely up to no fault of their own that suddenly their event does not exist… and they could have been training for that for 4–6 years and suddenly it’s gone” Neil (Con).

#### Setbacks

Setbacks were reported to be inevitable and potentially damaging. Nonetheless, the mentally tough athlete was perceived to respond to setbacks effectively.

“…how do I do this… the whole realization started to build on my shoulders and I did go into a state of depression… when it gets built up and you are challenged constantly in everyday life, and in competition, and then it just gets to a point where you physically and mentally cannot handle any more negativity or setbacks” Eddie (Acq).

“…London [2012] ended terribly for him … He trained for 4 years of his life, he was top of his game… massive disappointment from a home games, home support, home crowd… he almost decided to retire… but [said] you know what I am going to stick at it, I have still got plenty of games left in me and then go to Rio and absolutely blitz it on the track on his bike Errmm… And I think just the way that he went about it and he just did it, you know, there were no excuses” Eddie (Acq).

#### Trauma and Recovery

For the Paralympic athletes who acquired a disability, the critical incident (injury, accident, illness) was initially described as a distressing incident but following the recovery process the traumatic experience was considered an opportunity of growth and self-development.

“…was a professional… before her car accident so she has lost a lot, but she has still come out and made herself a champion… I just think that it is amazing, I think that is what MT is and I kind of look at myself and I think well, I was born the way I am so I do not know any different…I do not know what it is like to be able bodied so this is life, and I just get on with it. To know what you’re missing out on, I think that must be something pretty hard to bounce back from” Emma (Con).

“…got his leg blown off and he reached that stage nearly dying, and then have to come all the way back from that and then dealing with having to learn to walk again with new legs and then getting into training as part of rehab and then progressing to the elite level and representing GB, I suppose you have to be fairly mentally tough to get through all of that, especially the early stages” Jamie (Acq).

#### Sustained Commitment

In-sport pressures and situations were considered to be a test of personal commitment. Eddie and Neil’s accounts also demonstrate non-sporting pressures which affects personal commitment levels such as funding and livelihood.

“…it got to 2014, I was in the European Championships and I got disqualified once again for not physically being able to comply with the rules, my disability was at a level that I could not comply with the rules that were stated, and obviously I had that disappointment and then I thought in London this was going to be the change in the rules, and then after 16 years of battling classification, rule changes, then I got to the stage in my life, well I thought, do I really want to go through another 4 years with not having a guarantee that it is going to happen again” Eddie (Acq).

“…the Paralympic games which is fundamentally the most important performance of their life because: ‘A’ it is the pinnacle of their career, but ‘B’ their livelihood depends on it in terms of future funding and potentially becoming a high profile public figure, sponsorship etc., everything that will come with success, so that in itself is a very highly pressurized situation that every 4 years we have this unique event” Neil (Con).

#### Development of Mind-Set and Perspective during Challenge

Dedication, patience and hard work appear to contribute to the development of a mentally tough mind-set.

“…setbacks are going to happen, and things are going to happen that they do not like, but they just need to be able to deal with that and they need to get into the mindset that it is going to be a very long, hard, slow process…you just need to be…very patient you know what I mean, because I was not a very patient person before I had my accident but it taught me to be patient because things don’t always happen the first time or happen the way that you want them to” Tom (Acq).

“…so if I think about a Paralympic athlete or somebody going through a difficult period in terms of suffering an injury and surviving, I would have no doubts that there is something in that process…I would be fairly certain that that process gives them something, I mean if you have been through the lowest point where your life is at risk, relatively speaking everything else probably just, it’s never easy but you have perspective and think that this is not that bad” Neil (Con).

Eddie describes a pragmatic perspective which embellishes mentally tough characteristics and a positive appraisal during challenge.

“You have just got to kind of think of it that, that has become the halfway mark, realize the mistakes that you made and think well I have now got another 4 years to prepare, he didn’t suffer any injury so the 4 years that he had training before London, they were still there, all that preparation, all that hard work and all of that determination and the hours that he put in on his bike, they just roll over for another 4 years” Eddie (Acq).

#### Failure

It was reported that experiences of failure in sport provided opportunities to develop inner resources to perform and cope better in the future situations.

“…I think again the athletes with a big chip on the shoulder… if they are told that they cannot do something or even if they don’t win then you can see something seriously bad is happening, again it’s because they think the world owes them something and it really annoys me” Emma (Con).

“…when I first lost it was like Arrrgghh, I am not as good as I thought I was, but actually, you take a lot more from it and there is always that argument of, you can take more from a loss that you can from the win…I think you have got to put them in negative situations, you have got to try and teach them how to deal with negativity within sport, you know you have got to teach them disappointment, teach them how to fail” Eddie (Acq).

#### Acceptance

Self-acceptance was an ever present factor and considered a facilitator for MT and self-development.

“…it is about getting used to being disabled first before you start, you may not be comfortable being disabled, which you might go off and do other stuff, some people might go off and drink and do stuff like that, and maybe partying all the time because that helps them forget about it but there is still a problem at and they are not used to being disabled” Jamie (Acq).

“…to be able to accept that this has happened and it is not going to change, your arm is not going to grow back, your spinal-cord is not going to reattach… And I think that’s, that can be a period of 6 months for some people and it could be longer for others… And you do see people probably going through different phases of that… but again if I was in that situation, the options are, there really is only one option, you carry on and do what needs to be done” Neil (Con).

### Support and Coping Resources

Mental toughness support and coping resources refer to thoughts, feelings and other processes that contribute and affect the attitude, behavior and functions of the Paralympic athletes. The most salient of these are presented below.

#### Social Support and Significant Others

Athletes with a congenital disability identified their upbringing as a contributor to MT. Athletes that acquired their disability also reported parental and family support.

“…people carried me places because I could not walk, just because they did not want to accept that I was in a wheelchair, they did not want to accept that I was different, because sitting in that wheelchair made me feel like I was different whereas if I was walking around with everyone, okay I look different but in my head I looked like everybody else” Emma (Con).

“…but I am guessing that it is about the support network, the structure, the family upbringing, I have always had a very supportive family around me, and if I had a tough time, they would encourage me to keep trying and I have had a very supportive coach who has always try to put a positive spin on things” Eddie (Acq).

#### External Shaping

Social and training groups were seen as facilitators of MT. The account below describes social connection and a sense of both acceptance and normality within those environments.

“…I think that my Mom and Dad’s inability to accept that I cannot, I won’t, I will never… I think that is kind of where it has come from because they were very tough to be kind, they say that the doctor said to just give me an independent life, I guess it has just carried on from there like whatever I wanted to do and however, unrealistic it seemed I have found a way to do it” Emma (Con).

“…when I lost my legs I started doing sport, that was almost a buffer for me to start getting over my disability. So when that little bubble burst and I stopped competing the whole realization of me having a disability started to come into effect… almost realizing that I was in a chair because I was no longer in the environment with all my peers that had disabilities and competing in an environment where nearly everybody was the same” Eddie (Acq).

#### Overcoming Problems

Overcoming problems was perceived as a life skill, one that is developed by sport and non-sporting problems in a supported environment.

“…trying to overcome the challenges in the sport, I think it prepares you better for everyday life… if there are any challenges that you come across, you would not know how to deal with them, and again I think why I mentioned about being in a group, if you do not know how to do something more than likely someone will know because they have already done it, and they will show you and that’s the way to get around it” Jamie (Acq).

“…getting angry about stupid little things…the professional footballer Rich had gone and I had to find a new me, invent a new me, not a new version, but a new Rich, and fortunately through sport and the work that I do now, I have managed to do that and I feel like it actually happened for a reason… whereas before I could not see a reason” Rich (Acq).

#### Social Comparisons

Determination, self-belief, mind-set and perspective were developed by constantly making self-evaluations and comparisons to others. Dave’s account demonstrates a rational process that provides perspective in an accepting environment where disability is embraced and is the ‘norm.’

“I think that being surrounded by varying degrees of disability athletes all striving to be the best in the world as well, I would not say that it is easier to overcome, but I would say that it is easier to understand where you are at… you have got guys beside you with spinal cord injuries, and then you have got yourself and you are all striving in the same environment and all working as hard as you possibly can, all with different strengths and weaknesses” Dave (Acq).

“…if you went to a commercial gym, I would say that 99.9% of the time I am the only disabled person, whereas in the EIS gym, 90% of the time there about seven or eight of us training and it’s useful to feed off each other in a way that you know, the level of and you can give each other some stick” Dave (Acq).

#### Reflective Practice

Reflection on experiences was seen as an important preparation for future challenges, enabling athletes to respond more effectively in the future. Evaluations of personal feelings and memories were used by Dave to develop his inner resources.

“…I had been in hospital for about 6 months and I was in bed… I spilt the urine bottle that they gave me on the bed, I just literally broke into tears. I thought I am never going to get out of this bed and I think that that was the absolute lowest time I’ve ever had in the whole of my life… I just lost the plot and from that point I thought this is ridiculous and I am never going to feel like this, I lost it and from every other point in my life, I have been really calm… from that one moment I sort of built it back” Dave (Acq).

“…but that is me practicing reflective practice…it is probably more a case of looking at your own performance to see what you have done right and what you have done wrong… probably being self-critical on yourself, have I given it everything, have I shied away from certain aspects… am I using excuses?” Eddie (Acq).

## Discussion

This study had two related but different aims, initially focusing on MT conceptualization and then on MT development. As such, this discussion is presented in two sections, firstly focusing on conceptualization of MT in a Paralympian context, and then on MT development in that context. The findings relating to the first aim are relevant to MT conceptualization in general in that they expand the current literature on the conceptualization of MT across contexts. However, the findings related to the second aim prove useful beyond the immediate context, and are relevant to the development of MT in general. Our findings indicate that MT in Paralympic athletes is seen in terms of particular characteristics, cognitions and cognitive strategies. Some of these are particular to the specific challenges faced by this group of athletes; others are similar to those discussed in the general MT literature. The development of MT in this group involved exposure to a number of demanding sporting and non-sporting experiences including life changing trauma, these potentially damaging experiences proved to be beneficial when supported by the application of appropriate cognitive strategies and support systems.

### Conceptualization of MT in Paralympian Athletes

Relating our findings to previous research and theory was complex because the results appear to provide evidence partially supporting several conceptualizations of MT. Several distinguishing MT characteristics emerged that are in agreement with previous literature, and these characteristics were reported to be present during everyday challenges, stressors and adversities that Paralympians face both in and out of sporting environments (Cowden et al., 2014; Gucciardi et al., 2015b). The Paralympians perceived that their ability to overcome difficult situations was linked to their experience of the physical and mental challenges related to their disability. When considering the behaviors and characteristics of MT in Paralympians, the participants’ associated high levels of determination and persistence as an enabler for overcoming trauma, challenge and adversity, are similar to those found in able bodied athletes (Collins and MacNamara, 2012). The Paralympians’ approach to anticipated day-to-day life challenges and training programs were systematically recalled and suggest a very optimistic mind-set, positive self-belief and the employment of more emotion-focused strategies rather than the less effective problem-focused strategies. Methodical and pragmatic thinking patterns were described by the Paralympians as being essential for them to be self-organized and self-prepared, and for providing them with a higher sense of independence. These findings are in line with some hardiness literature that suggests hardy people perceive stressful circumstances as normal provocations to development (challenge), manageable (control), and worth investing in (commitment) (Maddi, 2004; Maddi and Harvey, 2006).

All participants interviewed experienced physical pain that accompanied their impairments and the secondary conditions related to these. These included tiredness and exhaustion, emotional regulation, feeling a lack of purpose outside of sport, and a lack of self-acceptance. These findings are similar to previously published work associated with this athletic population (e.g., Macdougall et al., 2016). The development of hardy and resilient characteristics and the Paralympians’ ability to cope with emotional and physical pain was linked to their exposure to constant pain and the associated emotional stress. Hardiness in Paralympians was described as a positive psychological component and emerged as a combination of interrelated attitudes (a cognitive/emotional amalgam) and interaction approaches (action patterns). This provides the Paralympians with determination, motivation, and strategies for turning imposed stresses from potential disasters into regular growth opportunities, and confirm previous findings (Maddi and Harvey, 2006). The result of this process was the creation of an, ‘I just have to live with it’ attitude, something that can be aligned to adversity-and-growth-related learning (Sarkar et al., 2015). Reoccurring perceptions of independence and autonomy were associated with the ability to endure higher levels of pain, performance and training stress, as well as the maintenance of motivation. Furthermore, a process of balancing conflicting identities, self-reflection, reframing and the motivation to live as ‘normal’ and as independent a life as possible, were seen to reduce the effect of stressful situations, and this category of adaptive outcomes have been referred to as ‘pragmatic coping’ (Salim et al., 2016). Consequently, athletes considered by participants as demonstrating lower levels of MT were described as being heavily dependent on others to regularly complete tasks for them and therefore removing the adversity stimulus required for the development of mentally tough characteristics.

Our findings reported a number of reasons for striving for the sense of independence which was interpreted as a sense of control, and produced feelings of autonomy, competence, satisfaction and achievement. Athlete representation in the public media was contrary to the Paralympians’ sense of identity and more globally it was evident that certain advertising campaigns were not in sync with Para-athletes philosophy or indeed the event itself. Specific exposure during Paralympic games and associations with ‘Super Human’ titles was seen as detrimental. The Para-athletes thought that such titles portrayed them as being ‘different’ to ‘human,’ and one of the driving factors for continuously enduring demanding situations and to be an elite athlete was linked with ‘normalization’ and to positively influence public perception. Engagement in activities that demonstrate athletic ability was seen to validate competence and was linked to improved psychological functioning and motivation levels, something that offers support for self-determination theory (SDT), specifically the surviving component which refers to effectively overcoming both major adversities as well as minor stressors in the ongoing pursuit of goals (Mahoney et al., 2014). Para-athletes viewed their mastery in sport as an opportunity to celebrate ability and project positive self-images in the hope of changing societal perceptions and frequently emphasized that appropriate comparisons should be made with other elite athletes such as Olympians.

The classification process is unique to Para-sport and was described as being a highly stressful in-sport factor with a perceived threat to athlete funding, selection and success. Clear links were made between a potential loss of funding and consequentially a loss of independence. The intent of standardizing competition levels was seen to provide levels of adversity that were uncontrollable, problematic, could not be prepared for and a threat to athlete identity. Similarly, classification was perceived to be a potential barrier to performance with event regulations and competition criteria offering a level of in-sport adversity. Participants felt that classification had been catastrophic to less mentally tough athletes and the disruption had resulted in them quitting. The more mentally tough athletes perceived the need to be non-accepting of potential constraints imposed by classification criteria and constantly required perseverance and problem solving skills.

In summary, the mentally tough Paralympian has been conceptualized as possessing a number of characteristics such as optimism, pragmatism, hardiness and resilience. The possession of these characteristics results in the athletes demonstrating superior coping skills, specifically when dealing with mental and physical pain. During physical and mental challenge associated with personal disability, Paralympians demonstrate determination, independence and autonomy. This approach to challenge is created by a group of distinct cognitions such as non-acceptance of limitations, a process of normalization and acceptance. Mentally tough Paralympians are considered to use a number of cognitive strategies to optimize function and performance as required.

### Development

Our findings suggest that deep self-reflection is a process that Para-athletes, and more specifically athletes with acquired disabilities, deemed as being crucial in developing MT. The process of acceptance and understanding physical limits was felt to be important in contributing to an athletic perspective, positive appraisals and goal achievement. Critical incidents (traumatic episodes) were initially described as catastrophic and life shattering. However, as the Para-athletes went through the process of acceptance, the traumatic experience was reframed to offer opportunity as professional athletes, ambassadors and mentors. Many athletes suggested that they would not ‘change their situation’ and the acquirement of the disability merely changed their goals. Furthermore, reflection on their personal traumatic experience has produced high levels of self-awareness, self-actualization and confidence in their ability to overcome demanding situations and moments of high challenge. Injury related growth has been reported to produce a realistic perspective and psychologically based performance enhancements such as an increased sense of MT, improved confidence and a commitment to training as a result of recovering (Roy-Davis et al., 2016; Salim et al., 2016). Despite the many situations where non-acceptance of limiting factors (potential obstacles) have been linked with MT in Paralympians, there is a consensus that acceptance of personal and individual disability, and specifically body awareness (e.g., recognition of physical limits) is important for initiating personal growth and development. Sport related injuries have previously been found to provide a traumatic experience whereby internal resources (e.g., personality, coping styles, knowledge, prior experience, and perceived social support) and external resources (i.e., cultural scripts, physical resources, time, and received social support) enable injured athletes to transform their injury into an opportunity for growth and development (Crawford et al., 2014; Roy-Davis et al., 2016). We believe that our findings add support to the sport injury related growth model. In addition, Para-athletes who acquired their disabilities through traumatic and potentially life-threatening injuries, also relates to Janoff-Bulman’s (2010) theory of shattered assumptions. Interpretations of the accounts demonstrated the ‘shattering effect’ their acquired disability had on their assumptive world (e.g., goals, beliefs, assumptions), with initial distress and depression followed by rebuilding and adopting new worldviews and personal growth both in and out of sporting environments (Crawford et al., 2014).

All Paralympians reported that interactions with particular social groups had contributed to the development and maintenance of MT. In relation to the MT characteristics reported in this paper, family members were associated with coping and transformational strategies, life-skills, problem solving and developing perspectives, which is similar to previous findings (e.g., Gucciardi et al., 2009). Specifically athletes with congenital disabilities described early and ongoing experiences of family members assisting them with their appraisal and to perceive the world as accessible and challenging rather than threatening, something that aligns to previous research findings (Mahoney et al., 2014). The Paralympians identified specific and significant situations whereby autonomy-support and a tough but supportive environment was seen as necessary to facilitate MT. Again, this is not unique to Paralympians, and has been highlighted previously (Tawse et al., 2012; Mahoney et al., 2014). Our findings revealed that Para-athletes with acquired disabilities reported a definite and deliberate focus on developing their inner resources in response to situational demands and they were able to experience personal growth. Additional to personal growth, vivid memories and reflections of traumatic experiences were used as a coping strategy whereby the current demands were compared to personal and potentially life threatening situations from their past. These evaluations enabled them to reframe a potential performance threat into a lesser challenge to overcome and they were able to experience positive affect (e.g., gratitude, optimism, interest), which led them to invest their time and effort into their physical and social resources, something also previously identified in Para-sport athletes (Macdougall et al., 2016; Salim et al., 2016). Engagement in such environments supported athlete identity and reinforced a mentally tough mind-set. Significant others and more experienced athletes were regarded as a key facilitators of MT by sharing knowledge of the sports and their disability, peer mentoring helped improve activities of daily living and enhanced the life satisfaction of others (Tawse et al., 2012).

### Lessons Learned

What can able-bodied athletes and coaches learn about developing MT from these athletes? Certainly the findings highlight the importance of having a positive attitude, taking on even the harshest of challenges rather than avoiding them, and being prepared to draw on all available resources, internal and external, to do this. These findings provide support for a number of previous theories and findings including PTG, athlete development, cognitive processing, affective engagement that has led to positive change and learning of the individuals adaptive resources (Tedeschi and Calhoun, 2004b; Bonanno and Mancini, 2008; Collins and MacNamara, 2012; Savage et al., 2016). Therefore we recommend that coaches and support staff should provide problem based scenarios such as potential event/ sport related situations (purposeful, problematic and realistic such as loss of equipment, late to venue or unfair officiating) and the use of consequences in training and competition to disrupt practice/performance. Overcoming disruptive events and distractions is required to enhance problem solving and coping skills. These findings imply that athletes should aim to view challenges with realistic optimism, use more emotion-focused strategies when dealing with difficult situations, and coaches should aim to gradually expose athletes to highly demanding physical and mental situations rather than shielding them from them to help develop superior coping skills.

In addition to the implications posited for athletes and coaches, we feel that there are implications for sport psychologists in general, and those working in the Para-sport environment in particular. The complex socially interconnected and nuanced internal and external environment the athletes in this study describe, point to the need for a holistic approach to psychological interventions in order to help athletes develop the reflective skills, self-knowledge and self-awareness required to navigate the world described. This highlights a need for sports psychologist to give serious consideration to approaches that focus on the person in context, and not just the athlete/performer. As such, we suggest sport psychologists explore alternative approaches in addition to those advocated by the dominant cognitive-behavioral paradigm, including humanistic and existential approaches (see Ronkainen and Nesti, 2017).

#### Limitations and Future Research

Although we attempted to focus on the conceptualization of MT in Paralympians, data indicated that athletes had experienced a variety of complex and multifaceted physical and mental disabilities and perceived ‘development opportunities.’ This is a limitation of our study, and in the future it may be important to focus more specifically on either athletes with congenital or acquired disabilities. Similarly, the classification of the disabilities and sport types could also vary participants’ perceptions of MT. Given the nature and differences between sport specific demands it may be difficult to generalize the findings given that MT has been difficult to conceptualize in able-bodied sport (Gucciardi et al., 2009). While we would encourage researchers to continue to seek greater clarification of the conceptualization of global MT and develop appropriate and valid measures relating to it conceptualization, we also recognize the value of taking an idiographic approach to MT. A personal construct approach (Kelly, 1955) would allow the use of individualized repertory grids to capture MT and changes in MT from both an in individual athlete and a coach’s perspective.

## Conclusion

To conclude, findings revealed that Paralympic athletes’ perceptions of MT can be seen as a combination of characteristics (determination, defiance, pragmatic, optimistic, resilient, self-belief and independence and autonomy), cognitions (normalization, sense of escape, non-acceptance of constraints, influence perception and connection) and cognitive strategies (rational thinking, goal setting, pain management and control). The development of MT requires a series of formative experiences (challenge, classification, setbacks, critical incident, trauma and recovery, sustained commitment, development of mind-set and perspective during challenge, failure, and acceptance), combined with support and coping resources (social support and significant others, external shaping, social support, overcoming problems, social comparison and reflective practice). Para-athletes have experienced a major life trauma, and experienced PTG, sports injury related and adversity-growth opportunities that require various coping resources and support systems to be developmental. Our findings highlight the association between the adaptive development of personal characteristics by overcoming physical and mental setbacks over a sustained time period. Overall, the findings suggest that athletes in general would benefit from exposure to highly demanding situations in a supportive environment to develop mentally tough characteristics and behaviors and to develop personalized cognitive strategies.

## Ethics Statement

The study protocol received approval from the ‘Research Ethics Committee of Newman University.’ The athletes’ participation was voluntary and written informed consent was obtained from each individual prior to data collection.

## Author Contributions

Conception and design of study: AP and TM; acquisition of data: AP; analysis and/or interpretation of data: AP and TM; drafting the manuscript: AP; revising the manuscript critically for important intellectual content and style: AP and TM; approval of the version of the manuscript to be published: AP and TM.

## Conflict of Interest Statement

The authors declare that the research was conducted in the absence of any commercial or financial relationships that could be construed as a potential conflict of interest.
